# Supplementary material for: Fibroin Delays Chilling Injury of Postharvest Banana Fruit via Enhanced Antioxidant Capability during Cold Storage
Source: Metabolites. 2019 Jul 23;9(7):152. doi: 10.3390/metabo9070152 (PMC6680957; doi:10.3390/metabo9070152)
Supplement: Supplementary file 1 [file metabolites-09-00152-s001.pdf]

Table S1

Primers for qRT-PCR analysis in peel of banana fruit

Gene	Accession No.	Forward primer (5′ to 3′)	Reverse primer (5′ to 3′)
POD	Ma11_t22010.1	GTGGACGAGATGGTGACGCTTTC	CCGCTTCGCCAGGTTCTTGTAG
SOD	Ma02_t04310.1	TGTTGTTGTTCTTGGTGGCAGTGA	AGCATGGCGATTGTCATCTTCAGG
APX	Ma05_t16920.1	CGGTGGAGAAGGCGAAGAGGAA	TGGTAGAAGTCAGCGTAGGTCAAGA
CAT	Ma08_t13920.1	TGCTTCCAGTAAATGCTCCCAAGTG	TTCTCACGCCTTCCAGTAACAACAG
GPX	Ma01_t04640.1	GCAGGAACCAGGAAGTAATGAGGAG	AGCATAGCGGTCCACCACATGA
GR	Ma07_t15360.1	CAGGCTGTTGGTGTTGAGGTTGAT	GCTGCTCGCTGAGACCTACTACT
MT1	Ma01_p10480.1	TGGACGAGAAGATGGTCAACTCTGA	GCAGGAGCAGCTAGATCCACACT
MT3	Ma03_p16510.1	GACAAGAGCCAGTGCGTGAAGAA	ACCACACTTGCACATGCCATCAT
GRX	Ma07_t13430.1	AGGATGCTGCATGAGCCACG	ACCGACGAAGAGGGTGGGAA
TRX	Ma02_t19350.1	TGTGGTCCTTGCCGTATGATTGC	CTGCCTCAGGAAGATGAAGGTTGG
Actin	Ma03_g21050	TGGTATGGAAGCCGCTGGTA	TCTGCTGGAATGTGCTGAGG
